# Use of Praziquantel as an Adjuvant Enhances Protection and Tc-17 Responses to Killed H5N1 Virus Vaccine in Mice

**DOI:** 10.1371/journal.pone.0034865

**Published:** 2012-04-18

**Authors:** Qiang Zou, Yanxin Hu, Jia Xue, Xiaoxu Fan, Yi Jin, Xianghua Shi, Di Meng, Xianzheng Wang, Congcong Feng, Xiaoping Xie, Yizhi Zhang, Youmin Kang, Xiaoxuan Liang, Bing Wu, Ming Wang, Bin Wang

**Affiliations:** 1 Key Laboratory of Medical Molecular Virology of MOH and MOE, Fudan University Shanghai Medical College, Shanghai, China; 2 State Key Laboratory for Agro-Biotechnology, College of Biological Science, China Agricultural University, Beijing, China; 3 College of Veterinary Medicine, China Agricultural University, Beijing, China; The University of Hong Kong, China

## Abstract

**Background:**

H5N1 is a highly pathogenic influenza A virus, which can cause severe illness or even death in humans. Although the widely used killed vaccines are able to provide some protection against infection via neutralizing antibodies, cytotoxic T-lymphocyte responses that are thought to eradicate viral infections are lacking.

**Methodology/Principal Findings:**

Aiming to promote cytotoxic responses against H5N1 infection, we extended our previous finding that praziquantel (PZQ) can act as an adjuvant to induce IL-17-producing CD8^+^ T cells (Tc17). We found that a single immunization of 57BL/6 mice with killed viral vaccine plus PZQ induced antigen-specific Tc17 cells, some of which also secreted IFN-γ. The induced Tc17 had cytolytic activities. Induction of these cells was impaired in CD8 knockout (KO) or IFN-γ KO mice, and was even lower in IL-17 KO mice. Importantly, the inoculation of killed vaccine with PZQ significantly reduced virus loads in the lung tissues and prolonged survival. Protection against H5N1 virus infection was obtained by adoptively transferring PZQ-primed wild type CD8^+^ T cells and this was more effective than transfer of activated IFN-γ KO or IL-17 KO CD8^+^ T cells.

**Conclusions/Significance:**

Our results demonstrated that adding PZQ to killed H5N1 vaccine could promote broad Tc17-mediated cytotoxic T lymphocyte activity, resulting in improved control of highly pathogenic avian influenza virus infection.

## Introduction

Avian A/H5N1 Influenza A virus, emerged as a cause of human disease in recent years that is associated with high mortality and it poses a major pandemic threat [1]–[3]. Vaccination has its advantages over other approaches for limiting potential pandemic influenza outbreaks. Although, the widely used killed H5N1 vaccine is helpful by eliciting a neutralizing antibody response in chickens [4], protection against H5N1 infection in humans is likely to require eliciting both humoral and cellular virus-specific responses. Eliciting cytolytic T lymphocyte responses may be particularly important since this has been demonstrated to eradicate viral infections [5]–[8]. The induction of broad cytotoxic T lymphocyte responses to killed H5N1 vaccine is an urgent and challenging issue.

Adjuvant could be added into the killed vaccine to elicit a robust and broad cellular immune response [9], [10]. Till now, alum and MF59 are the only two adjuvants approved by the U.S. Food and Drug Administration (FDA) for use in influenza vaccines [9], [11], but both are limited by minimal induction of cell-mediated immunity [12]–[16]. Novel adjuvants are therefore needed to enhance cellular immunity for these killed viral vaccines. Praziquantel (PZQ) has an excellent record of safety in treating *Schistosomiasis* infections [17], and recently we demonstrated that PZQ could enhance cellular responses to HBsAg DNA vaccination [18], [19]. Therefore we investigate here whether PZQ could enhance cellular immune responses to the killed H5N1 vaccination.

In this study, we demonstrated that by using PZQ with a killed H5N1 vaccine mice could be better protected against a lethal-dose challenge with H5N1 virus. This protection was strongly associated with elicitation of more IL-17-producing cytotoxic CD8^+^ T cells (Tc17) when PZQ was used with the killed H5N1 vaccination. This is the first report to show that PZQ has great potential as an adjuvant to induce strong CD8^+^ T cell-immunity with killed H5N1 vaccine.

## Results

### PZQ as adjuvant induced a high level of cytolytic response

**Figure 1:**
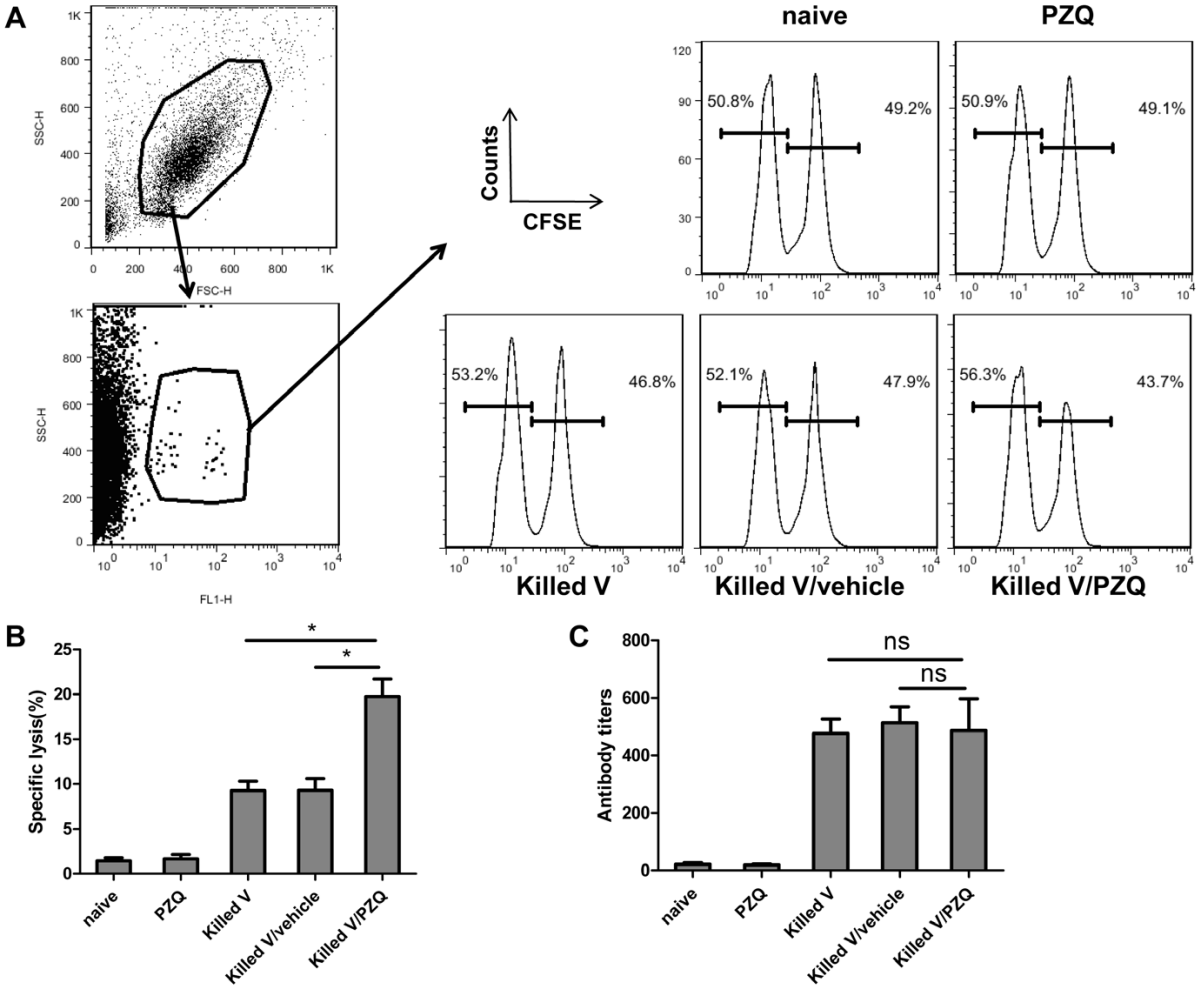
In vivo cytotoxic responses and antibody responses in immunized mice. C57BL/6 mice were immunized with 0.1 µg killed H5N1 vaccine in 100 ul of delivery vehicle, with vehicle alone, with vehicle containing PZQ (0.5 µg PZQ/0.1 µg antigen) or with vaccine in vehicle containing PZQ. (A) Analysis of in vivo cytotoxic lysis on day 7 after primary immunization. (B) The percentage of specific lysis summarized as the means of the three independent experiments. (C) Antibody levels in serum collected on day 14 after immunization and detected by ELISA. Data shown are representative from three independent experiments. *, p<0.05. ns, p>0.05. Killed V stands for the killed H5N1 vaccine and vehicle stands 7.5% ethanol in saline solution.

Since virus specific cytotoxic T lymphocyte activity is indispensable for controlling viral infections [7], [8], we first examined whether adding PZQ to the killed H5N1 vaccine (0.5 µg PZQ/0.1 µg antigen) could induce higher levels of nucleoprotein (NP)-specific cytolytic responses. Mice were immunized a single time and in vivo cytotoxic activity was assayed 7 days later. Compared to the groups that were immunized with the killed vaccine alone or the killed vaccine with vehicle, the highest level of antigen-specific lysis was observed in the mice immunized with the killed vaccine plus PZQ (Figure 1A–B).

Since antibody production is also an important arm of immune response, we also tested antibody titers of serum samples taken 14 days after the vaccination. There were no differences between the groups (Figure 1C), suggesting that PZQ did not affect the humoral response.

### PZQ promoted Tc17 cell activation

**Figure 2:**
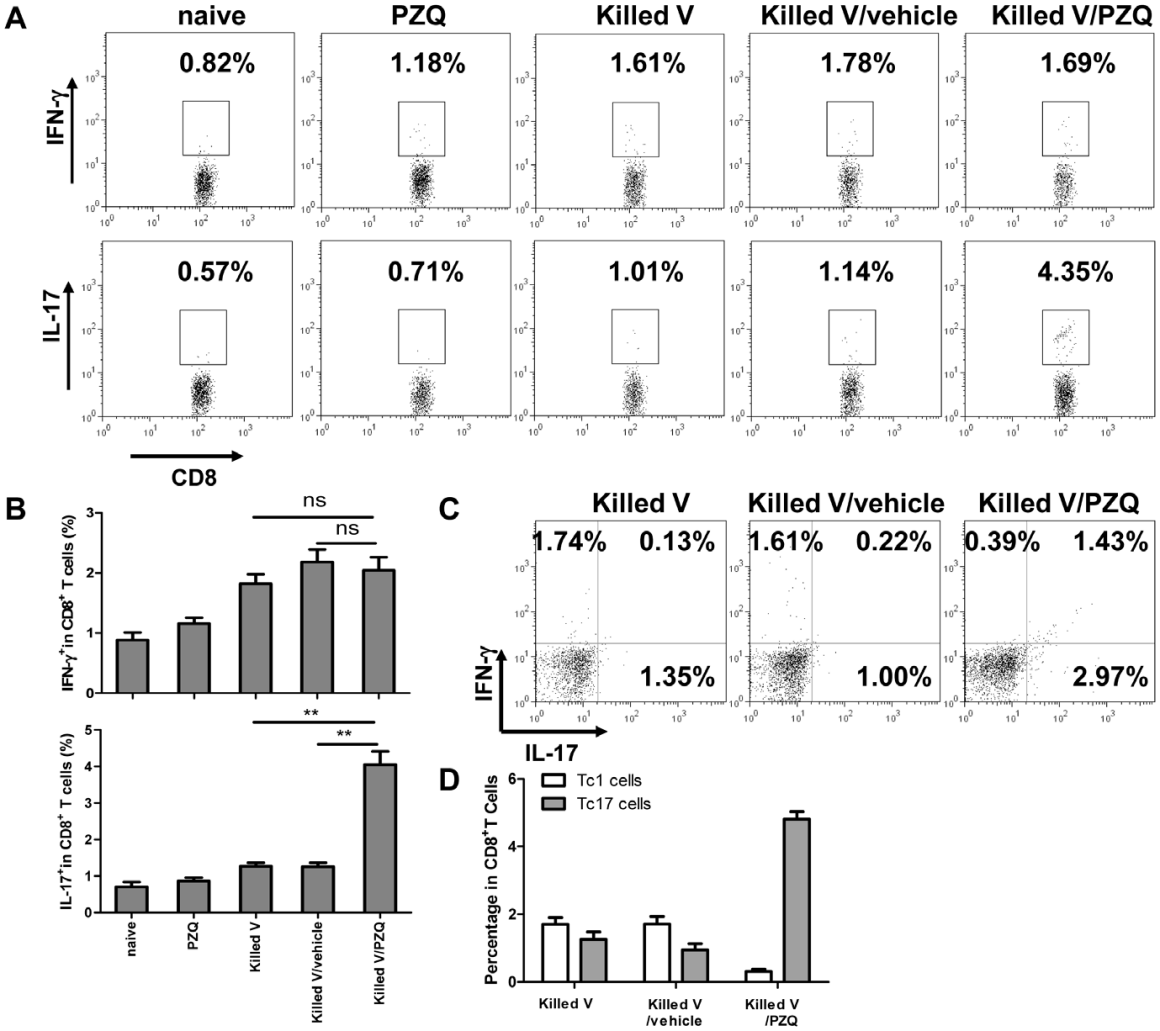
Analysis of antigen-specific cytokine productions in CD8^+^ T cells. Splenic cells were isolated from C57BL/6 mice on day 7 after immunization and stimulated with NP peptide for 6 h in culture. (A) Intracellular staining for IFN-γ, IL-17 and CD8^+^ was analyzed by FACS. (B) The percentages of positive-stained cells are summarized as the means from three independent experiments. (C) Co-staining for IFN-γ and IL-17 in CD8^+^ T cells. (D) The percentages were shown as the means of three independent experiments. **, p<0.01. ns, p>0.05.

Because both IFN-γ-producing CD8^+^ T cells (Tc1) and IL-17-producing CD8^+^ T cells (Tc17) could contribute to the cytolytic response [20]–[22], we sought to determine which subset of T cells were the main effectors. Splenocytes were isolated 7 days after immunization and stimulated with NP peptide (ASNENMETM) in vitro before they were stained for intracellular IFN-γ, IL-17 or both and assayed by flow cytometry. As shown in Figure 2A–B, the expression of antigen-induced IL-17, but not IFN-γ in CD8^+^ T cells was significantly higher in the mice immunized with killed vaccine plus PZQ compared to other groups. This was confirmed by the result of double staining shown in Fig. 2.C–D, which revealed that 2.3% of such splenocytes were IL-17-positive/IFN-γ-negative (Tc17) and only 0.4% were IFN-γ-positive/IL-17-negative (Tc1). In addition, there was an increased appearance of splenocytes staining positive for both cytokines (1.4%). The results suggested that PZQ facilitated Tc17 cell differentiation.

### Tc17 cells were the main effectors for cytotoxic responses

**Figure 3:**
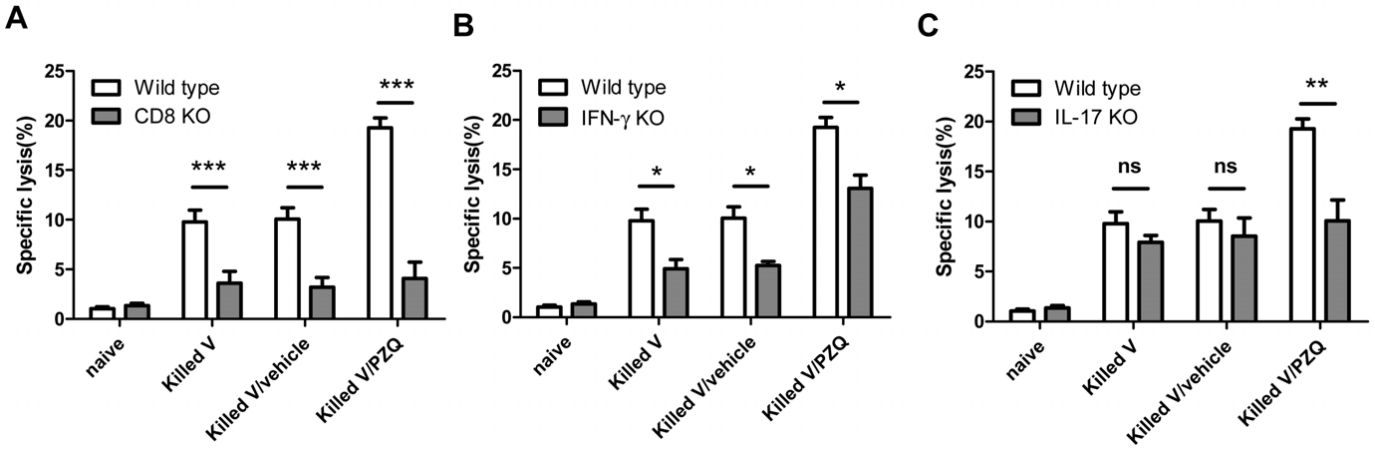
In vivo cytotoxic responses in immunized wild-type, CD8KO, IFN-γKO and IL-17KO mice. Mice were tested at day 7 after immunization and the percentage of specific lysis is summarized. (A) In vivo cytotoxic lysis was tested in the wild-type and CD8KO mice. (B) In vivo cytotoxic lysis in IFN-γ KO mice. (C) In vivo cytotoxic lysis in IL-17KO mice. The same data from wild-type mice is shown in each panel for reference.*, p<0.05. **, p<0.01. ***, p<0.001. ns, p>0.05. KO stands for knockout.

To examine whether the induced IL-17-positive were the major effectors for the cytotoxic activity, we employed CD8, IFN-γ or IL-17 KO mice in the cytotoxicity assays. The cytotoxic response was almost entirely eliminated in the CD8 KO mice (Fig. 3A) and the enhancing effect of PZQ on the response was reduced in the IFN-γ and eliminated in the IL-17KO (Fig. 3B–C). The results demonstrated that PZQ as adjuvant can enhance cytolytic response via activation of IL-17-positive Tc17 cells.

### PZQ enhanced protection against H5N1 lethal challenge

**Figure 4:**
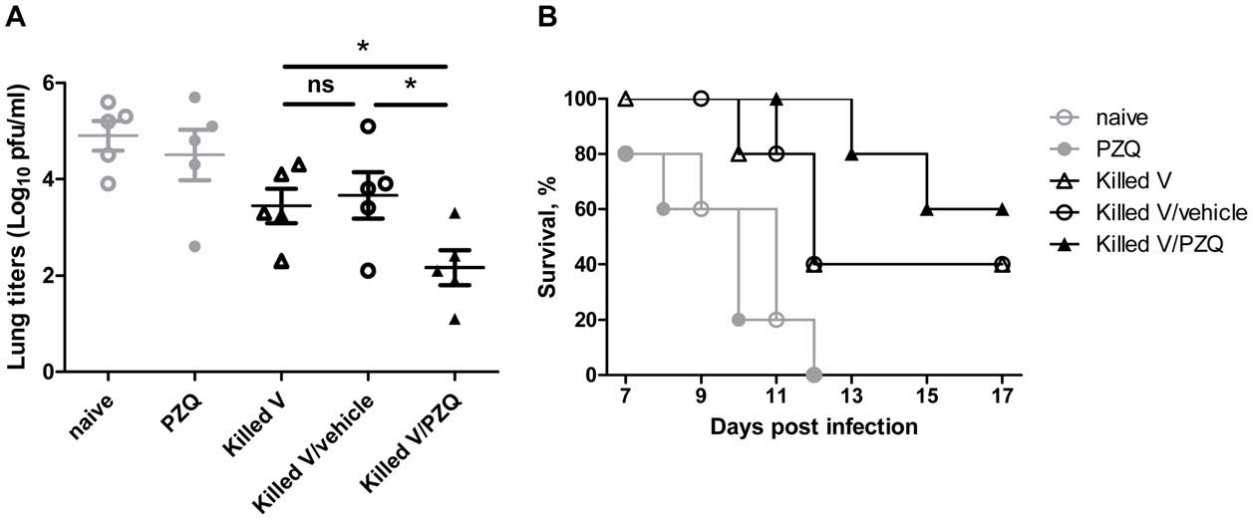
Protection against a highly pathogenic avian influenza virus H5N1 infection. Mice were challenged one month after vaccination (0.5 µg PZQ/0.1 µg antigen). (A) Viral loads in the lungs on day 7 after virus infection. (B) Survival curves. Data are analyzed from three separate experiments and each had 5 animals. *, p<0.05.

To determine whether vaccination by the killed viral vaccine plus PZQ could protect animals against lethal H5N1 viral challenge, mice were vaccinated with a low dose (0.1 µg) of killed H5N1 vaccine with or without PZQ (0.5 µg PZQ/0.1 µg antigen) and challenged on week four after the vaccination. The killed vaccine alone or the killed vaccine with vehicle reduced the viral load in the lungs. Significantly greater reduction of viral load was observed in the mice immunized with the killed vaccine plus PZQ (Fig. 4A). Furthermore, the survival rate of mice vaccinated by the killed vaccine plus PZQ was 60% versus the 40% of those vaccinated with the vaccine without PZQ (Fig. 4B). The results were consistent with the higher level of cytotoxic responses conferring greater protection.

**Figure 5:**
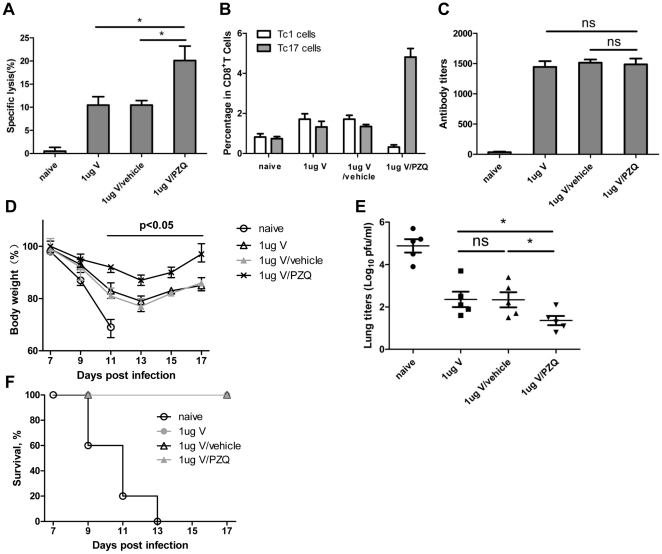
Effect of adding PZQ to high-dose (1 µg) killed H5N1 vaccine. Mice were immunized with high-dose killed H5N1 vaccine (0.5 µg PZQ/1 µg antigen). (A) In vivo cytotoxic lysis assay performed on day 7 after primary immunization. (B) The percentage of Tc cells at day 7. (C) Antibody levels in sera collected on day 14 after immunization and assayed by ELISA. (D) Weight change of surviving mice and (E) viral loads in the lungs. Mice were infected x days after immunization and viral load was measured 7 days later. (F) Survival curves. Data are analyzed from three separate experiments and each had 5 animals. *, p<0.05. ns, p>0.05. 1 µg V stands for 1 µg killed H5N1 vaccine.

We next tested if higher dose of killed influenza vaccine could provide a greater protection. We used 1 µg of the killed vaccine with or without the PZQ as adjuvant (0.5 µg PZQ/1 µg antigen). In agreement with results above, a significantly greater cytotoxic response and a higher percentage of Tc17 cells were induced by 1 µg of killed vaccine plus PZQ compared with the vaccine without PZQ. The levels of antibody were again not different between the groups (Fig. 5A–C). After lethal challenge, mice vaccinated by the killed vaccine with or without PZQ were 100% protected. However, mice vaccinated by the killed vaccine plus PZQ showed delayed weight loss and the lowest viral loads in their lungs compared with other groups (Fig. 5D–F). The data supported a strong correlation between a high level of Tc17-mediated cytolytic response and protection.

### Tc17 cells are involved in protection against H5N1 virus infection

**Figure 6:**
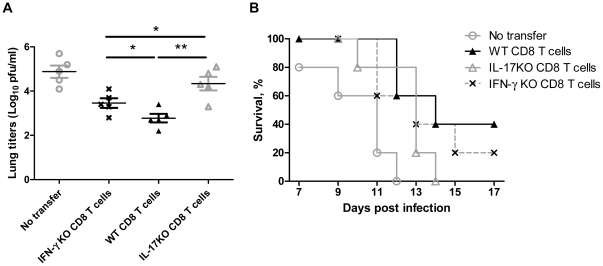
Adoptive transfer of sensitized CD8^+^ T cells delays the development of virus infection. Each mouse received 5×10^6^ cells and was then challenged immediately with a lethal dose of H5N1 virus. (A) Viral loads in the lung of mice assayed on day 7 after infection. (B) Survival curves. Data are analyzed from three separate experiments and each had 5 animals. *, p<0.05. **, p<0.01. WT CD8 T cells stands for CD8^+^ T cells from wild type mice immunized with killed vaccine plus PZQ. IFN-γ KO CD8 T cells stand for CD8^+^ T cells from similarly immunized IFN-γ KO mice. IL-17KO CD8 T cells stands for CD8^+^ T cells from similarly immunized IL-17KO mice.

To test the importance of a Tc17-mediated cytolytic response during the H5N1 viral infection, primed CD8^+^ T cells from vaccinated mice were used for adoptive transfer of protection. The cells were isolated on day 7 from IFN-γ KO, IL-17KO or wild-type C57BL/6 mice that had been vaccinated with 1 µg killed H5N1 vaccine with or without PZQ (0.5 µg PZQ/1 µg antigen). CD8^+^ T cells were purified to 96–98% of purity and adoptively transferred intravenously into naïve syngeneic mice at 5×10^6^ cells per recipient mouse immediately before H5N1 viral challenge. Naïve mice that did not receive cells developed high viral loads and were all dead by day 12. Primed CD8^+^ T cells from IFN-γ KO or wild-type mice could provide some degree of protection with delayed deaths and lower viral loads. However, CD8^+^ cells from IL-17KO mice gave very little reduction in either death rate or viral load (Fig. 6A–B), suggesting a protective role of Tc17 during the viral infection. These data suggest that both Tc1 and Tc17 contributed to the control of H5N1 viral infection, but the Tc17 apparently contributed more.

Such cell-mediated immune responses can be through recognition of conserved epitopes of internal proteins of the influenza virus such as NP and PA [23]–[25], consequently they may protect animals from heterologous viral challenge. To test this, we vaccinated mice with 1 µg killed H1N1 vaccine plus PZQ or 1 µg killed H5N1 vaccine plus PZQ (0.5 µg PZQ/1 µg antigen) and challenged with H5N1 virus. Cross-protection did not occur as shown in Figure S1A. In addition, the primed-CD8^+^ T cells induced by either vaccination were isolated on day 7 after vaccination and transferred to normal syngeneic mice, which were then challenged with H5N1 virus. The protective effect during virus infection was then measured by the survival rate and the virus titer in the lungs. Although the killed H5N1 vaccine plus PZQ-primed CD8^+^ T cells protected against H5N1 challenge, the corresponding killed H1N1 vaccine plus PZQ-primed CD8^+^ T cells did not (Fig. S1B–C). The results suggested that the killed vaccine with PZQ as adjuvant did not support cross-protection.

## Discussion

Virus-specific cytotoxic T lymphocyte activity has been demonstrated to be essential for controlling influenza viral infections [7], [8], yet the widely used formalin-inactivated vaccines do not induce CTL responses specific for influenza virus antigens [26]. In this study we found that a single immunization of mice with the killed H5N1 viral vaccine plus PZQ was able to induce IL-17-secreting CD8^+^ T cells, many of which also secreted IFN-γ. Vaccination with the killed vaccine plus PZQ also gave significantly greater protection to the mice against a lethal virus challenge infection. There was reduction of virus loads in the lung tissues and prolonged survival. This protection was associated with the Tc17 cell activation since an adoptive transfer of these cells reduced viral loads and prolonged survival. Thus our data show, for the first time, that protective virus-specific Tc17 cells can be primed in vivo during vaccination. How PZQ affects the T cell responses in such a specific way remains to be elucidated.

Previous studies demonstrated that inactivated vaccines against influenza viruses induced antibodies specific for the virus but not effective CTL responses [26], [27]. However, in the present study, formalin-inactivated vaccines against H5N1 virus alone induced CTL responses. This situation has been described in several previous publications. Particularly, it was reported that formalin-inactivated H5N1 whole virus particles were effective for induction of both cytotoxic T-lymphocyte and antibody responses against highly pathogenic avian influenza viruses in vivo [28]. This induction of CTL presumably goes through the so-called cross-presentation pathway. Although the pathway is present in APCs, adjuvants may facilitate entry of antigens into it. Another report showed that heat-aggregated influenza virus induced significant CTL responses [29]. It is tempting to speculate that inactivated vaccines might stimulate dendritic cells to indirectly activate CTL precursors because ultraviolet-inactivated influenza virus induced IFN-α production in plasmacytoid dendritic cells [30]. Further investigation is needed to clarify this point.

Although Tc17 cells have been shown to promote inflammation during the development of experimental autoimmune encephalomyelitis and influenza infection [22], [31], [32], their function was not clear. Several reports have indicated that IL-17-producing cells that were not cytotoxic [33]–[35], whereas others proved they had cytotoxic phenotype [20], [22], [36]. In addition, in vivo transfer of in vitro-derived Ag-specific Tc17 cells has been shown to clear lethal doses of influenza [36], [37]. Our present finding of an association between increased Tc17 response and increased protection that could be adoptively transferred indicates that Tc17 cells may be critical for anti-viral activity.

It has been reported by others that although most Tc17 cells produced IFN-γ, a small part of the Tc17 cell population was IFN-γ negative in some individuals [20], [22], [38]. Acquiring the Tc1 phenotype (IFN-γ production) was reported to be not critical for the anti-viral activity of Tc17 cells [22] but the biological function of IFN-γ in Tc17 cells is not clear. In our study, PZQ induced both populations of Tc17 cells, IFN-γ negative cells and IFN-γ positive cells, during killed H5N1 vaccination. However, our studies in KO mice indicated that when depleted of IFN-γ the Tc17-mediated cytotoxic and anti-viral responses were impaired, indicating that acquiring the Tc1 phenotype may be necessary for the development of Tc17-mediated cytotoxic and anti-viral activity.

Effector Tc1 cells, the classical cytotoxic T cells that produce IFN-γ and TNF-α, have been demonstrated to control lung inflammation during acute influenza virus infection [39]–[41]. It was striking that here NP366–374 peptide-specific Tc1 cells were not expanded, and yet IFN-γ positive Tc17 cells were expanded. Till now, little work has been done to clarify whether IFN-γ inhibits or promotes the development of Tc17 and it is possible that Tc1 cells could produce IL-17 to convert to Tc17 cells in the presence of PZQ. Further studies are needed to clarify it.

There is considerable interest in the development of universal vaccines that can afford protection against different subtypes of influenza A virus [6], [42]. In contrast to antibody responses, CTL differentially recognize and respond to influenza virus of different subtypes, mainly recognizing epitopes derived from conserved proteins [43]–[45]. It has been shown that CTL specific for seasonal influenza virus can cross-react with H5N1 virus [45], [46], and the cross-reactive CTL responses also afford heterosubtypic immunity [47]. However, in the present study, we found that there was no cross protection against H5N1 virus infection by CTL specific for H1N1 viruses. We speculate that there are some mutations of epitopes derived from internal proteins between the two variants of influenza virus. Next, we will analyze the CTL responses to different epitopes of internal viral proteins, comparing mice immunized with either killed H5N1 or killed H1N1 vaccine plus PZQ.

Both antibodies and CTL specific for H5N1viruses are critical for protection against H5N1 virus infection. This is partly because avian influenza virus strains replicate both in the upper respiratory tract and in the lung and intestine [48], [49]. Interestingly, the inclusion of PZQ did not enhance the antibody responses that were obtained by immunizing with either 0.1 µg or 1 µg H5N1 inactive vaccine. It appears that PZQ preferentially promoted cell-mediated immunity, not antibody responses, and we need further experiment to understand the mechanism.

In summary, we show for the first time that the addition of PZQ as an adjuvant to killed H5N1 vaccine induces strong Tc17-mediated CTL and enhances protection against a highly pathogenic avian influenza virus infection. PZQ may therefore represent a promising chemical adjuvant for killed vaccines and provide a novel strategy where the induction of a broad CD8^+^ T cell immunity is required.

## Materials and Methods

### Reagents and Animals

Praziquantel (NCPC, Hebei, China) was first dissolved in ethanol to 6.7% and subsequently diluted to 0.5% with the saline solution. The vaccine delivery vehicle was therefore 7.5% ethanol in saline solution. Fluorescently labeled anti-mouse monoclonal antibodies for flow cytometry were purchased from BD Pharmingen (San Diego, CA, USA). Influenza virus nucleoprotein (NP) NP366–374 peptide representing the CD8^+^ T-cell epitope (ASNENMETM) was synthesized by GL Biochem Co., Ltd. Adult female C57BL/6 mice at 8–10 weeks of age were purchased from Animal Institute of Chinese Medical Academy (Beijing, China). IFN-γKO (B6.129S7-Ifng^tm1Ts^/J) mice were purchased from the Jackson Laboratory (Bar Harbor, Maine). CD8 KO mice (C57BL/6 background) and IL-17 KO mice (C57BL/6 background) were from kindly provided by Richard Flavell (Yale University School of Medicine, New Haven, CT). All animal protocols (#28482-rev1) were approved by the Animal Welfare Committee of China Agricultural University and housed with pathogen-free food and water under 12 h light-cycle conditions.

### Vaccines

Highly pathogenic avian influenza virus, (HPAIV, H5N1, A/Chicken/Henan/1/04) was maintained at BSL-3 facility (China Agriculture University, Beijing, China). Whole H5N1 chemically inactivated viruses was used as killed vaccine and was provided by Dahuanong Animal Health Inc. (Guangdong, China). The inactivated vaccine was prepared by treating the H5N1 virus (A/Chicken/Henan/1/04) with 0.2% formalin at 37°C for 24 h. The vaccine was tested for its immunogenicity in mice and inability to infect chicken embryos. H1N1 killed vaccine was kindly supplied by Zhejiang Tianyuan Bio-Pharmaceutical Co., Ltd. (Zhejiang, China). The protein content of the vaccines was determined with Pierce BCA Protein Assay Kit (Rockford, IL, USA).

### Immunization

The C57BL/6 mice were randomly divided into five groups (n = 10 each). The killed vaccines, at a dosage of 0.1 µg or 1 µg and with/without adjuvant (0.5 µg PZQ/0.1 µg or 1 µg antigen), injected intramuscularly. The volume of each dose was 100 µl.

### Virus challenge

Four weeks after the single dose of immunization, mice were challenged with a lethal dose (10 LD_50_) of mouse-adapted strain A/Chicken/Henan/1/04 (H5N1) by intranasal administration of 20 µl of the viral suspension. This infection caused death of all the unimmunized mice within 7–12 days.

### In vivo CTL assay

In vivo cytotoxic assay was performed using splenocytes from naïve C57BL/6 mice pulsed with 10^−6^ M NP peptide and labeled with a high concentration of CFSE (15 µM, CFSE^high^ cells) as target cells. A portion of the same splenocytes was labeled with a low concentration of CFSE (0.5 µM, CFSE^low^ cells) without peptide pulse as a non-target control. The target and control cells were mixed in a 1∶1 ratio and injected into immunized mice at 2×10^7^ total cells per mouse via the tail vein on day 7 after primary immunization. Four hours later lymph nodes and the spleens of injected mice were removed and the target and control cells were analyzed by their differential CFSE fluorescent intensities using a FACSCalibur (BD Biosciences, USA). Specific lysis was calculated using the following formula: ratio = percentage CFSE^low^/percentage CFSE^high^. Percentage specific lysis = [1−(ratio unprimed/ratio primed)] ×100.

### Detection of anti-H5N1 antibody

Serum anti-H5N1 antibody was measured with an enzyme-linked immunosorbent assay (ELISA). On day 14 after immunization, serum was collected. ELISA was performed in a 96-well polystyrene microtiter plate with reagents consisting of inactivated H5N1 virus vaccine. Following the blockage with 3% of BSA for 1 h, the plate was incubated with diluted mouse sera in each well. A second goat anti-mouse IgG Ab (Sigma,St. Louis, Mo) diluted 1,000 times was added. Ten milligrams of TMB tablet (Sigma,St. Louis, Mo) was dissolved by 0.025 M phosphate-citrate buffer and subsequently added to each well for color development. After the reaction was stopped by 2 M H_2_SO_4_, the plate was read with a plate reader at 450 nm/620 nm. The antibody titers were determined by the absolute ratio of OD values of post/naive sera at a cutoff ratio of 2.1.

### Flow cytometric analysis

Splenic cells were isolated on day 7 after the final immunization and stimulated with 10 µg/ml NP peptide in the presence of brefeldin A (5 µg/ml) for 6 h at 37°C and 5% CO_2_. Collected cells were fixed with 4% paraformaldehyde and permeabilized with 0.1% saponin (Sigma-Aldrich). For immunostaining of cytoplasmic IL-17 and IFN-γ and surface CD8, the appropriate concentrations of fluorescently labeled anti-mouse monoclonal antibodies were added to permeabilized cells for 30 min on ice followed by washing twice with cold PBS. Samples were analyzed by a FACSCalibur.

### Viral RNA determination

Total RNA was prepared from 10 mg lung homogenized and extracted in Trizol (Invitrogen) according to the manufacture's instruction. DNase I treated RNA (2 µg) was used to reverse transcribe into cDNA using a set of universal primers for influenza A virus [50] as following: forward primer, 5′-CGCAGTATTCAGAAGAAGCAAGAC-3′; reverse primer, 5′-TCCATAAGGATAGACCAGCTACCA-3′. Real-time PCR was performed to amplify the hemagglutinin (HA) gene of H5N1 influenza virus using SYBR® PrimeScript® RT-PCR Kit (TaKaRa Inc, Japan). The reaction was run on an ABI 7500 and data analysis was performed using 7500 software v2.0 (ABI). The copy number of the HA gene was calculated by using a HA-containing plasmid of known concentration as a standard.

### Adoptive transfer of CD8^+^ T cells

On day 7 after primary immuzation with 1 µg killed vaccine and PZQ, single-splenocyte suspensions were prepared from spleen of wild type, IFN-γ KO or IL-17KO mice. CD8^+^ T cells were isolated and purified using the MagCellect Mouse CD8^+^ T Cell Isolation Kit according to the manufacturer's protocol (R&D Systems, Inc., Minneapolis, USA). Purity of each cell preparation was 90–95%. The cells were adoptively transferred intravenously into normal C57BL/6 mice at 5×10^6^ per recipient mouse.

### Statistical Analysis

Results are presented as means ± S.E.M. Student's *t* test analysis was used for data analysis. A value of *p*<0.05 was considered to be statistically significant.

## Supporting Information

Figure S1 **Weak cross protection against H5N1 virus by CTL specific for H1N1 virus.** C57BL/6 mice were immunized with 1 µg killed H5N1 vaccine plus PZQ or 1 µg killed H1N1 vaccine plus PZQ (0.5 µg PZQ/1 µg antigen), and challenged with H5N1 virus one month later. (A) Survival curves. Alternatively, 7 days after immunization, CD8^+^ splenocytes were obtained and transferred to naive recipients that were immediately challenged with H5N1 virus. (B) Viral loads in the lung on day 7 after infection. (C) Survival curves. Data are analyzed from three separate experiments and each had 5 animals. CD8 T cells (H5N1/PZQ) stands for CD8^+^ T cells from C57BL/6 mice immunized with 1 µg killed H5N1 vaccine plus PZQ. CD8 T cells (H1N1/PZQ) stands for CD8^+^ T cells from C57BL/6 mice immunized with 1 µg killed H1N1 vaccine plus PZQ. *, p<0.05. ns, p>0.05.
(TIF)
