# Supplementary figures and images for: Use of Praziquantel as an Adjuvant Enhances Protection and Tc-17 Responses to Killed H5N1 Virus Vaccine in Mice
Source: PLoS One. 2012 Apr 18;7(4):e34865. doi: 10.1371/journal.pone.0034865 (PMC3329547; doi:10.1371/journal.pone.0034865)

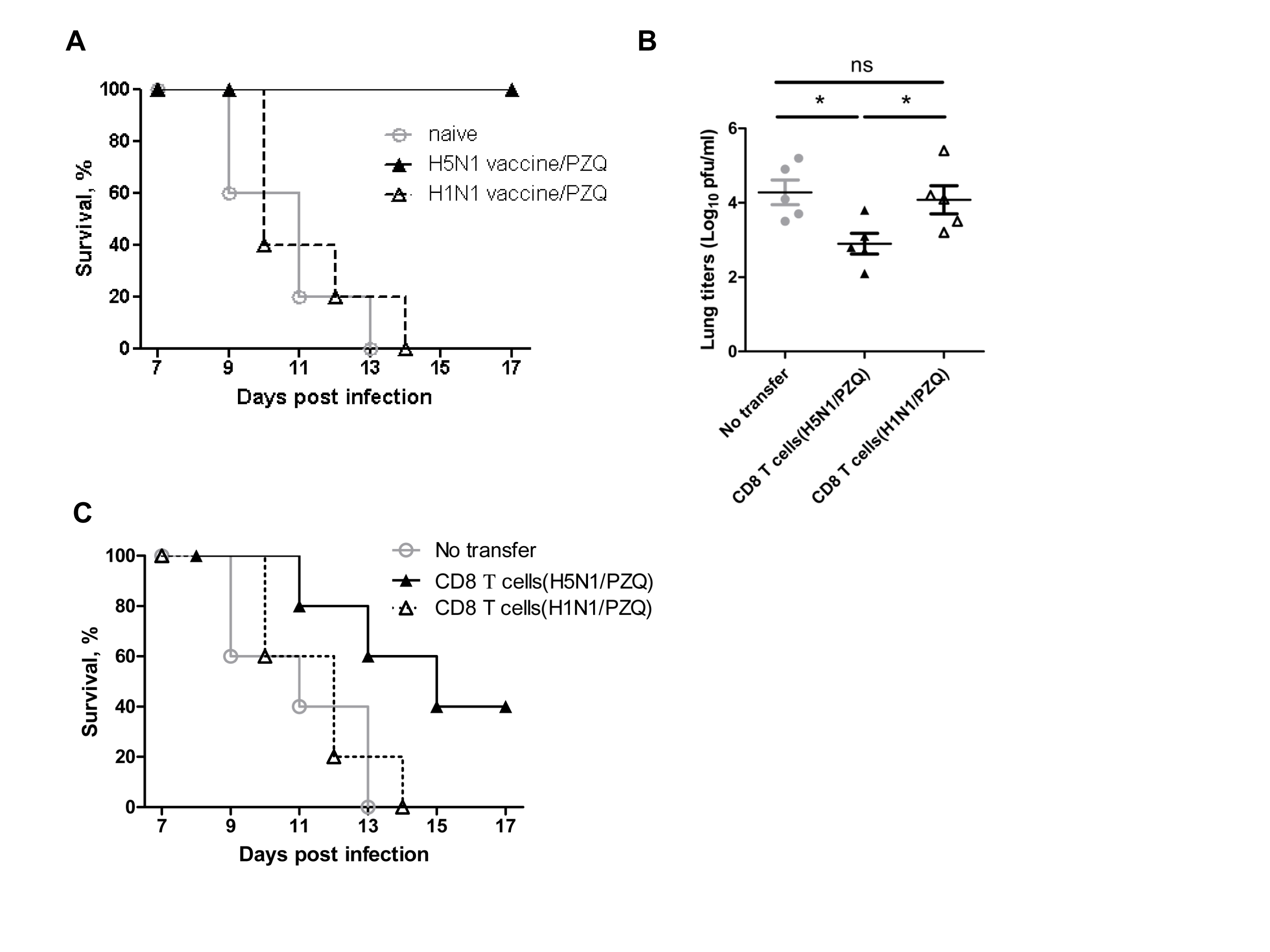

Supplement: Figure S1 — Weak cross protection against H5N1 virus by CTL specific for H1N1 virus. C57BL/6 mice were immunized with 1 µg killed H5N1 vaccine plus PZQ or 1 µg killed H1N1 vaccine plus PZQ (0.5 µg PZQ/1 µg antigen), and challenged with H5N1 virus one month later. (A) Survival curves. Alternatively, 7 days after immunization, CD8+ splenocytes were obtained and transferred to naive recipients that were immediately challenged with H5N1 virus. (B) Viral loads in the lung on day 7 after infection. (C) Survival curves. Data are analyzed from three separate experiments and each had 5 animals. CD8 T cells (H5N1/PZQ) stands for CD8+ T cells from C57BL/6 mice immunized with 1 µg killed H5N1 vaccine plus PZQ. CD8 T cells (H1N1/PZQ) stands for CD8+ T cells from C57BL/6 mice immunized with 1 µg killed H1N1 vaccine plus PZQ. *, p<0.05. ns, p>0.05. (TIF) [file pone.0034865.s001.tif]
